# Immune Senescence: Relative Contributions of Age and Cytomegalovirus Infection

**DOI:** 10.1371/journal.ppat.1002850

**Published:** 2012-08-16

**Authors:** Andrea Mekker, Vincent S. Tchang, Lea Haeberli, Annette Oxenius, Alexandra Trkola, Urs Karrer

**Affiliations:** 1 Division of Infectious Diseases and Hospital Epidemiology, University Hospital of Zurich, Zurich, Switzerland; 2 Molecular Life Science Graduate School, University of Zurich, Zurich, Switzerland; 3 Institute of Microbiology, Swiss Federal Institute of Technology (ETH) Zurich, HCI 4, Zurich, Switzerland; 4 Institute of Medical Virology, University of Zurich, Zurich, Switzerland; Oregon Health Sciences University, United States of America

## Abstract

Immune senescence, defined as the age-associated dysregulation and dysfunction of the immune system, is characterised by impaired protective immunity and decreased efficacy of vaccines. Recent clinical, epidemiological and immunological studies suggest that Cytomegalovirus (CMV) infection may be associated with accelerated immune senescence, possibly by restricting the naïve T cell repertoire. However, direct evidence whether and how CMV-infection is implicated in immune senescence is still lacking. In this study, we have investigated whether latent mouse CMV (MCMV) infection with or without thymectomy (Tx) alters antiviral immunity of young and aged mice. After infection with lymphocytic choriomeningitis virus (LCMV) or Vaccinia virus, specific antiviral T cell responses were significantly reduced in old, old MCMV-infected and/or Tx mice compared to young mice. Importantly, control of LCMV replication was more profoundly impaired in aged MCMV-infected mice compared to age-matched MCMV-naïve or young mice. In addition, latent MCMV infection was associated with slightly reduced vaccination efficacy in old Tx mice. In contrast to the prevailing hypothesis of a CMV-mediated restriction of the naïve T cell repertoire, we found similar naïve T cell numbers in MCMV-infected and non-infected mice, whereas ageing and Tx clearly reduced the naïve T cell pool. Instead, MCMV-infection expanded the total CD8^+^ T cell pool by a massive accumulation of effector memory T cells. Based on these results, we propose a new model of increased competition between CMV-specific memory T cells and any ‘de novo’ immune response in aged individuals. In summary, our results directly demonstrate in a mouse model that latent CMV-infection impairs immunity in old age and propagates immune senescence.

## Author Summary

Cytomegalovirus (CMV) persistently infects 50–90% of the human population. After primary infection, constant immune surveillance is required to prevent CMV-related disease. During ageing, increasing T cell resources are expended to keep CMV under control. Recent human studies have suggested that this investment may come at the cost of accelerated immune senescence, a condition describing the age-associated decline of the immune system's functionality. In the present study, we have developed a mouse model to directly investigate whether and how CMV-infection might impair immunity of aged individuals. We demonstrate that old mice with long-lasting CMV-infection are more susceptible to viral infections than old mice without CMV since their virus specific T cell response is suppressed. Contrary to the prevailing hypothesis we found no indication for a CMV-associated shrinking of the naïve T cell compartment. Instead, CMV-infection precipitated a massive expansion of memory T cells. Thus, we propose an alternative mechanism of CMV-enhanced immune senescence based on T cell competition between CMV-specific memory T cells and de novo generated T cell responses. In summary, we provide the first direct evidence that CMV-infection is indeed a propagating factor for poor immunity in the elderly.

## Introduction

Immune senescence, defined as the age-related alterations of the immune system, is associated with an increased incidence of infections, cancer, autoimmunity and a reduced efficacy of prophylactic vaccines [1]–[3]. Although all components of the immune system undergo age-related changes, the T cell compartment is most significantly affected by a quantitative and qualitative loss of naïve T cell diversity due to declining thymic output and increasing dysregulation of compensatory homeostatic mechanisms [4]–[8]. Therefore, ageing hosts have increasing difficulties to mount efficient primary T cell responses whereas memory maintenance and recall responses appear to be less affected [9]–[11].

Immune senescence is certainly a multifactorial process involving genetic, molecular, cellular and also environmental factors. Among the latter, Cytomegalovirus (CMV) infection has gained considerable interest in recent years as a potential propagator of human immune senescence [12]. First, two independent epidemiological studies have linked human (H)CMV-seropositivity with decreased overall survival of elderly [13], [14]. Second, a series of Swedish cohort studies of very elderly have identified a so called ‘immune risk profile’ (IRP), which was strongly predictive of all cause mortality. Importantly, HCMV-infection was one of the most important IRP-parameters [15]. Third, protective antibody titres after influenza vaccination were reduced in HCMV-positive compared to HCMV-negative elderly individuals, although this finding was not confirmed in a subsequent study [16], [17]. Together, these studies suggest that HCMV-infection may be associated with decreased immunocompetence of the elderly. However, it is unclear whether HCMV-infection is causally linked with accelerated immune senescence or whether it is just a marker for something else - like poor nutrition during childhood - since HCMV-infection is known to be associated with lower socio-economic resources [18]. Moreover, very little solid information is available about potential underlying mechanisms of HCMV-induced immune senescence. Immunologically, HCMV-infection is characterised by inducing very prominent T cell responses with the highest magnitude of all investigated persistent pathogens, and this response regularly occupies 20% and more of the total CD8^+^ T cell pool [19]. In addition, T cell responses seem to increase with duration of infection due to memory inflation both in mice and in humans [20]–[25]. This has fostered the hypothesis that these massive CMV-driven memory T cell expansions significantly accelerate the age-associated loss of naïve T cells which are indispensible for potent ‘de novo’ immune responses [26]–[28]. Others have speculated that HCMV-infection and HCMV-specific T cells maintain a proinflammatory cytokine milieu which may be suppressive for immunity in the elderly [29], [30].

Therefore, an animal model for the direct investigation of CMV-enhanced immune senescence would be extremely informative 1) to establish a clear link between CMV-infection and accelerated immune senescence, 2) to quantify the influence of CMV, 3) to elucidate potential viral and immunological mechanisms, and 4) to develop intervention strategies for its prevention. In this study, we have combined mouse (M)CMV infection with or without thymectomy (Tx) of young adult mice to generate a model for CMV-enhanced immune senescence. By performing Tx in mice we aimed to recapitulate the human situation of a relatively early decline of thymic T cell output [31]. In humans, thymic involution begins shortly after birth and only a minimal number of new thymic emigrants reach the periphery in adults, leading to a progressive loss of naïve T cell numbers and diversity during ageing [32], [33]. In mice, the thymus remains fully functional for prolonged periods of time in relation to their life expectancy [34].

Our results show that both MCMV-infection and Tx significantly decrease antiviral immune responses and protection of ageing mice. In contrast to Tx, which substantially reduced naïve T cell numbers over time, MCMV-infection did not measurably influence the absolute size of the naïve T cell pool. Instead, MCMV-infection induced a significant and long-lasting expansion of the total CD8^+^ T cell compartment by the massive accumulation of effector memory T cells (Tem). Therefore, we propose an alternative model of CMV-enhanced immune senescence based on increasing T cell competition. Overall, we present the first data from a mouse model to confirm that latent CMV-infection itself has a propagating influence on the development of immune senescence.

## Results

### Latent MCMV-infection is associated with impaired viral control and reduced virus-specific CD8^+^ T cell immunity in old mice

To test whether latent MCMV-infection has an influence on immune control of heterologous viral infections, mice were infected intravenously (i.V.) with 10^7^ plaque forming units (pfu) MCMV-Δ157 at the age of 6–8 weeks. MCMV-Δ157 is a mutant virus with a targeted deletion of the MCMV open reading frame (ORF) *m157* encoding a ligand for the NK cell activating receptor Ly49H [35]. After infection of Ly49H-positive C57BL/6 mice with MCMV-Δ157 NK cell stimulation and NK cell mediated early viral control were substantially attenuated [35]. Moreover, infection with MCMV-Δ157 was recently characterised by increased early antigen load, elevated levels of innate cytokines but preserved conventional dendritic cell (cDC) function leading to more robust antiviral CD8^+^ T cell responses which were indispensable for termination of productive MCMV-Δ157 infection [36]. Since we hypothesised that the magnitude of MCMV-specific T cell responses was involved in MCMV-enhanced immune senescence we performed all subsequent experiments using MCMV-Δ157 to enhance the probability of a measurable effect. Despite attenuated NK cell activity, C57BL/6 mice have usually cleared this virus below the limit of detection in all organs within 6–8 weeks indicating the establishment of viral latency (data not shown). Young or old mice with or without latent MCMV-infection were then infected with different, heterologous viruses and viral replication and antiviral CD8^+^ T cell responses were quantified.

**Figure 1:**
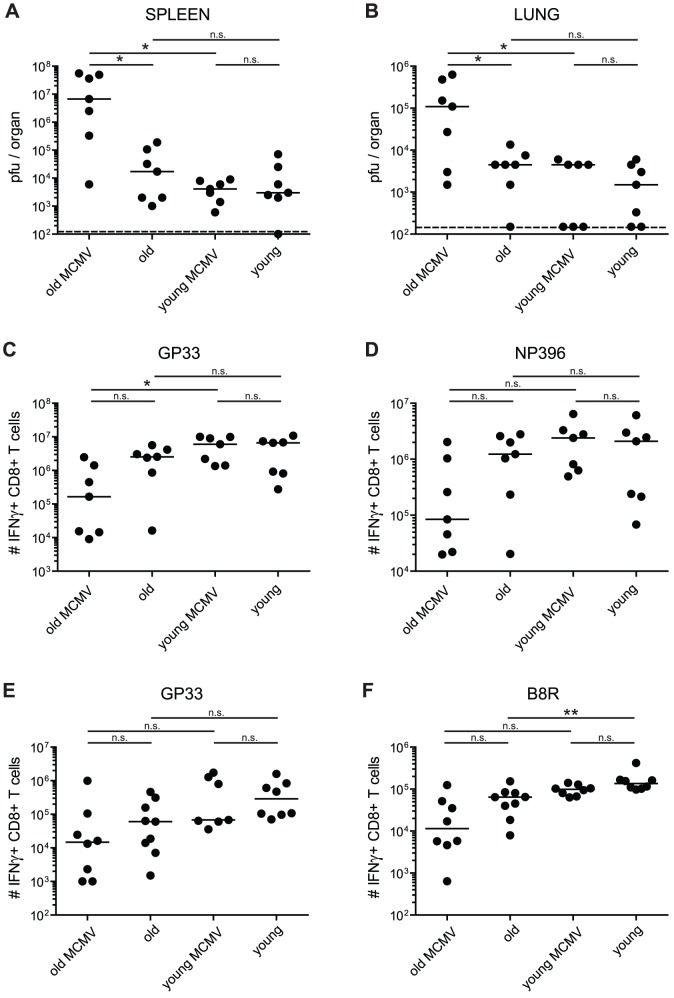
LCMV-clearance and LCMV- or VACV-specific CD8^+^ T cell responses in young and old mice with/without latent MCMV-infection. A–D: uninfected or MCMV infected (10^7^ pfu MCMV-Δ157 i.v.) young (4 or 6 months old; 2 or 4 months p.i.) and old (15 or 22 months old; 11 or 18 months p.i.) C57BL/6 mice were infected with 2×10^3^ pfu LCMV-WE i.v. Eight days after infection, LCMV titres were determined in spleen (A) and lung (B) by plaque forming assay and the virus-specific CD8^+^ T cell response was quantified by ICS in the lung for GP33-specific (C) and NP396-specific (D) CD8^+^ T cells. E, F: young (4 months old, 2 months p.i.) and old C57BL/6 mice (25 or 28 months old, 23 or 26 months p.i.) with and without latent MCMV-infection were infected with 5×10^6^ pfu VACV-GP i.p. At the peak of the response on day 6, GP33-specific (E) and B8R-specific (F) CD8^+^ T cells from the lung were quantified by ICS after *in vitro* re-stimulation with the respective peptide. Circles indicate total numbers of epitope specific CD8^+^ T cells of individual mice, horizontal lines show the medians of an individual group. Data are pooled from two independent experiments. ANOVA followed by Bonferroni post-analysis was used to determine significant differences between the displayed medians of each group (* *p*<0.05; ** *p*<0.01; ns = not significant). The dotted line corresponds to the detection limit of the assay. p.i., post infection.

First, we analysed viral control and T cell immunity after infection with lymphocytic choriomeningitis virus (strain WE; LCMV-WE), since LCMV elicits a very potent CD8^+^ T cell response, which is required for early viral control [37]. It has been demonstrated before that aged mice are less efficient in mounting protective LCMV-specific T cell responses [10]. Young (4 or 6 months old; 2 or 4 months after MCMV-infection) and old mice (15 or 22 months old; 11 or 18 months after MCMV-infection) with and without latent MCMV-infection were infected i.v. with 2×10^3^ pfu of the LCMV-WE. Eight days later, LCMV-titres were quantified in different organs (Figure 1A, B for lung and spleen; liver and kidney not shown) and the LCMV-specific CD8^+^ T cell response against two immunodominant epitopes derived from the viral glycoprotein (GP)33 and nucleoprotein (NP)396 was measured in the blood, spleen and lung by tetramer staining or intracellular cytokine staining (ICS) (Figure 1C, D for lung). As shown in figure 1A and B, young mice were able to control LCMV-replication within 8 days to levels between 1.5–3×10^3^ pfu/organ in spleen and lung, irrespective of latent MCMV-infection. In parallel, total numbers of GP33- and NP396-specific CD8^+^ T cells were comparable in lung (Figure 1C, D), spleen and blood (data not shown) in MCMV-infected and MCMV-naïve young mice. These results indicate that latent MCMV infection does not measurably influence LCMV replication or LCMV-specific CD8^+^ T cell responses in young mice. As expected, LCMV control was affected by age, since LCMV titres were between 3- and 6-fold higher in old compared to young mice although the difference did not reach statistical significance (*p = not significant (ns)*; Figure 1A, B). In parallel, total numbers of LCMV-specific CD8^+^ T cells were between 2- and 3-fold lower in old compared to young mice in all organs tested (*p = ns*; Figure 1C, D for lung; spleen and blood not shown). These rather small age-associated differences in viral control and LCMV-specific T cell responses are comparable with earlier findings from Kapasi et al [10]. More importantly, LCMV control and LCMV-specific T cell responses were additionally reduced in old MCMV-infected compared to old MCMV-naïve mice. Overall, LCMV titres were between 25- and 1,150-fold higher in old MCMV-infected compared to old MCMV-naïve mice in spleen (6.7×10^6^ vs. 1.7×10^4^ pfu/organ, *p<0.05*), lung (1.1×10^5^ vs. 4.5×10^3^ pfu/organ, *p<0.05*), liver (5.8×10^7^ vs. 5×10^4^ pfu/organ, *p<0.05*) and kidney (6.7×10^4^ vs. 2.7×10^3^ pfu/organ, *p<0.1*), and between 40- and 2,300-fold higher compared to young mice, independent of MCMV infection (spleen *p<0.05*, lung *p<0.05*, liver, *p<0.05*, kidney *p<0.1*; Figure 1A, B; data not shown). In parallel, GP33- and NP396-specific CD8^+^ T cell responses were about 15-fold (lung) or 3-fold (spleen) in old MCMV-infected compared to old MCMV-naïve mice (*p = ns*; Figure 1C, D; data not shown). Although absolute numbers of LCMV-specific CD8^+^ T cells were measurably reduced in old mice with or without latent MCMV-infection, we did not identify relevant functional or phenotypic differences between LCMV-specific CD8^+^ T cells from these four groups of mice with respect to cytokine production (IFNγ, TNFα), cytotoxicity measured by degranulation (i.e. CD107a-upregulation) or expression of activation markers like CD43 (not shown).

To corroborate these findings in a different viral model, similarly treated four groups of young (4 months old) and old mice (25 or 28 months old) with and without latent MCMV-infection were prepared and infected intraperitoneally (i.p.) with 5×10^6^ pfu of Vaccinia virus (VACV) recombinant for LCMV-GP (VACV-GP). Six days later, antiviral CD8^+^ T cell responses were quantified in the blood, spleen and lung and VACV titres were measured in the ovaries. Unfortunately, VACV replication could not be reliably assessed in the ovaries of old and old MCMV-infected mice since we observed massive differences (up to 10^5^-fold) of VACV-replication in left and right ovaries of individual mice and within groups of mice. We suspect that VACV-replication in ovaries was heavily influenced by individualised onset of reproductive arrest. However, in young mice, latent MCMV-infection had no measurable influence on VACV-GP clearance (data not shown).

Concerning the CD8^+^ T cell responses, the previous findings using LCMV were confirmed after VACV-infection since we recorded similar trends of attenuated T cell responses in old and latently infected old mice. In young and young MCMV-infected mice, comparable numbers of CD8^+^ T cells specific for two out of three immunodominant VACV-derived epitopes and for the recombinant LCMV-derived epitope GP33 were recorded at the peak of the response on day 6 in lung (Figure 1E, F and Figure S1A, B) and spleen (data not shown). CD8^+^ T cell numbers were reduced by a factor of 4 to 14 in old compared to young mice indicating that ageing also affected T cell immunity after VACV-GP infection (B8R, A3L, A8R: *p<0.01*; GP33: *p = ns*). Compared to old naïve mice latent MCMV-infection in old mice further reduced VACV-GP induced CD8^+^ T cell numbers in the lung and spleen by a factor of 2.5 to 6 (*p = ns* for all epitopes and organs; Figure 1E, F and Figure S1A, B for lung; spleen not shown). Overall, these data indicate that both ageing and latent MCMV-infection have a quantitatively comparable influence on antiviral T cell immunity in old mice. Both effects are modest but consistent and additive in old mice with latent MCMV-infection.

### MCMV control and MCMV-specific CD8^+^ T cell responses are not affected by thymectomy

Thymic involution is considered to be a major factor for T cell based immune senescence in humans [38] but in mice, thymic involution is considerably delayed in relation to their life expectancy [31], [34]. Therefore, we next investigated whether Tx of mice at the age of 4–5 weeks would influence and enhance the development of immune senescence, particularly in the context of latent MCMV infection. We first tested whether MCMV control and MCMV-specific immune responses were comparable between Tx and non-Tx wild type (wt) C57BL/6 mice. Control of lytic MCMV replication was similar in Tx and wt mice (Figure S2) and all mice were free of detectable virus in all organs tested and had thus established latent MCMV-infection within 6–8 weeks after MCMV-Δ157 infection (not shown).

**Figure 2:**
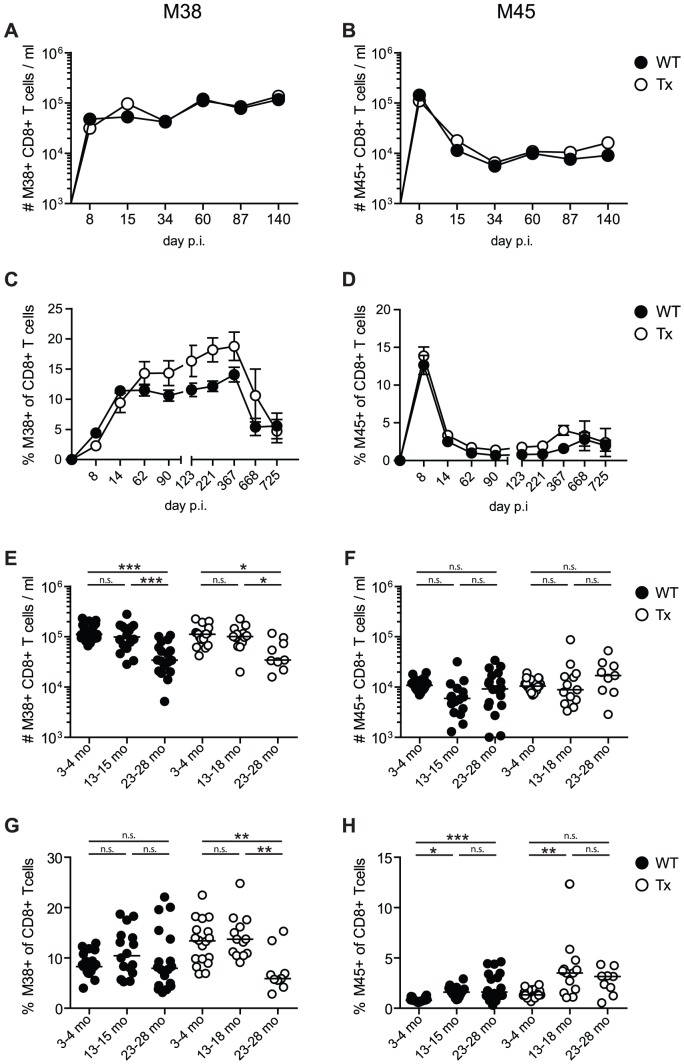
Long-term analysis of MCMV-specific CD8^+^ T cell responses in thymectomised and non-thymectomised mice. C57BL/6 mice were thymectomised at age of 4–5 weeks. Two to 3 weeks later, Tx mice (open circles) and non-Tx wild type mice (wt, closed circles) were infected with 10^7^ pfu MCMV-Δ157 i.v. The MCMV-specific CD8^+^ T cells were quantified in the blood by tetramer staining and analysed by polychromatic flow cytometry using beads for direct measurements of total numbers. A–D: longitudinal analysis of the total number of M38- (A) and M45-specific (B) CD8^+^ T cells in the blood. Longitudinal analysis of the frequencies of M38- (C) and M45-specific (D) CD8^+^ T cells in the blood at different time points after MCMV infection. Circles indicate the mean of 8–11 (A, B) or 5–7 (C, D) mice per group, error bars indicate the SEM. Figure E–H: cross-sectional analysis of the total numbers (E, F) and the frequencies (G, H) of M38- (E, G) and M45-specific (F, H) CD8^+^ T cells in the blood of young (3–4 months), middle-aged (13–18 months) and old C57BL/6 mice (23–28 months). Data of the cross-sectional analysis were pooled from three independent experiments. ANOVA followed by Bonferroni post-analysis was carried out for calculating significance (* *p*<0.05; ** *p*<0.01; *** *p*<0.001; ns = not significant). p.i., post infection.

We also compared the MCMV-specific CD8^+^ T cell responses longitudinally in wt and Tx mice, focussing on M45-specific CD8^+^ T cells, which usually display a conventional T cell kinetic of expansion, contraction followed by low level and stable memory T cell numbers, and M38-specific CD8^+^ T cells which display the phenomenon of memory inflation [20], [22], [24]. As shown in Figure 2A, C, memory inflation of M38-specific T cells occurred mainly during the first three months after infection and was not influenced by Tx. The apparent increase of M38-specific T cell frequencies in Tx-mice (Figure 2C, G) was caused by a loss of total CD8^+^ T cells after Tx and disappeared in the analysis of total T cell numbers (Figure 2A, E). Interestingly, despite the abolished recruitment of new naïve T cells in Tx mice, M38-specific CD8^+^ T cells underwent a similar degree of memory inflation in Tx and wt mice (Figure 2A, C). This confirms earlier findings, that thymic replenishment of the naïve T cell pool and the amount of recent thymic emigrants is not crucial for memory inflation [38], [39]. Very advanced age was accompanied by an impressive loss of the inflated M38-specific CD8^+^ T cell population, both in normal and Tx mice (Figure 2C, E, G). This could indicate that a large proportion of M38-specific memory T cells were reaching their proliferative limit (Hayflick limit) after >16 months of MCMV-infection at the age of about 20 months and that the naïve M38-specific precursors were exhausted as well. The conventional T cell kinetic of expansion, contraction and stable memory of the M45-specific CD8^+^ T cell population was not significantly influenced by Tx (Figure 2B, D, F, H). M45-specific cells were stably maintained at low levels well into senescence with a slight trend for increasing numbers at very old age (Figure 2D, F) suggesting that these cells and their naïve precursors were not exhausted.

### After immunisation with virus-like particles cellular and humoral immunity is significantly impaired by ageing and thymectomy but only marginally by latent MCMV-infection

**Figure 3:**
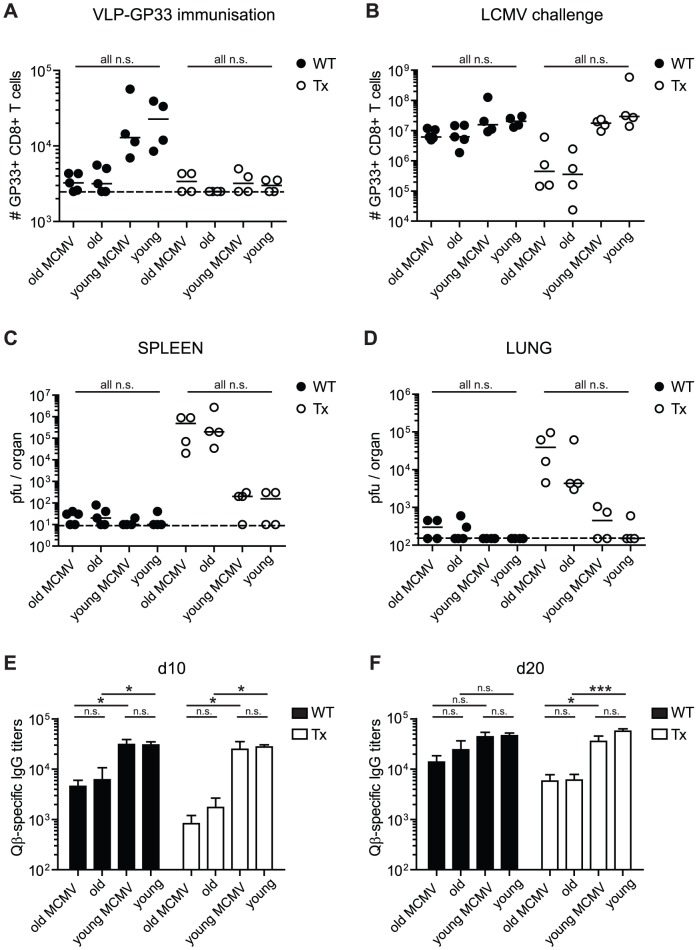
Immunogenicity and efficacy of VLP-immunisation against LCMV-challenge in young and old mice with/without Tx or latent MCMV-infection. Young (5 months old, 3 months p.i.) and old C57BL/6 mice (18 months old, 15 months p.i.) with or without Tx and latent MCMV-infection were immunised with 10 µg VLP-GP33 s.c. Thereafter, the GP33-specific CD8^+^ T cell response was measured on day 7 (A) by tetramer staining and the Qb-specific IgG titres were quantified by ELISA on day 10 (E) and day 20 (F) in the blood. On day 21, all mice were challenged with 2×10^3^ pfu LCMV-WE i.v. Eight days after LCMV-challenge the GP33-specific CD8^+^ T cell response was determined by ICS in the lung (B) and LCMV-titres were determined in the spleen (C) and in the lung (D) by plaque assay. Circles indicate individual mice, horizontal lines represent the median (A–D). Vertical bars show the mean of an experimental group, error bars correspond to the SEM (E, F). The dotted line shows the detection limit of the assay. 4–5 mice per group were included. ANOVA followed by Bonferroni post-analysis was preformed to test for significant differences (* *p*<0.05; ** *p*<0.01; ns = not significant).

To test and compare the impact of age, Tx and MCMV-infection on cellular and humoral immunity after a potent vaccination approach, mice were immunised with bacteriophage Qβ-derived virus-like particles (VLP) coupled to the LCMV peptide GP33 (VLP-GP33) and adjuvanted with CpG-oligonucleotides [40]. Before immunisation, mice were thymectomised (or not) at the age of 4–5 weeks and 2–3 weeks later, they were infected (or not) with 10^7^ pfu MCMV-Δ157. Three or 15 months later, all mice were immunised with VLP-GP33 s.c. to induce a LCMV-GP33-specific CD8^+^ T cell response. On day 7 after VLP-immunisation, GP33-specific CD8^+^ T cell numbers were clearly reduced in the blood of old mice and of all Tx mice (Figure 3A). Quantitatively, the effect of 12–15 months older age was equivalent to Tx at young age and the two effects seemed to be additive in old Tx mice which displayed the strongest reduction of GP33-specific T cell numbers after priming with VLP-GP33 (Figure 3A) and more clearly after secondary challenge with LCMV-WE (see below, Figure 3B). Latent MCMV-infection did not have a measurable influence on VLP-induced T cell immunity, since GP33-specific CD8^+^ T cell numbers were already at the limit of reliable detection in old and in Tx mice (Figure 3A).

We next investigated, whether reduced priming efficacy after VLP-GP33 immunisation was associated with decreased T cell mediated antiviral protection after a challenge with LCMV-WE. On day 21 after VLP-GP33 immunisation, these eight groups of mice were infected intravenously with 2×10^3^ pfu LCMV-WE. Eight days later, the GP33-specific CD8^+^ T cell response was analysed in several organs, including the lung (Figure 3B). Despite reduced priming efficiency, the GP33-specific recall CD8^+^ T cell response after LCMV-challenge was comparable between aged and young mice in non-Tx animals. In addition, young Tx mice mounted similar T cell responses to non-Tx mice. Compared to all other groups, aged Tx animals had between 10- and 15-fold reduced numbers of GP33-specific T cells after LCMV-challenge. However, in none of the tested groups latent MCMV-infection had a measurable influence on the GP33-specific recall CD8^+^ T cell response.

We also measured LCMV-titres in spleen, liver, lung and kidney of these mice. Consistent with the size of the CD8^+^ T cell responses, all non-Tx mice had nearly cleared LCMV from all organs tested including old and old/MCMV-infected mice (Figure 3C for spleen, 3D for lung; filled symbols). When comparing LCMV titres after VLP-GP33 immunisation (Figure 3C, D; filled symbols) with LCMV titres without immunisation (Figure 1A, B), it is apparent that VLP-GP33 immunisation was highly protective and was able to largely overcome the age- and MCMV-associated impairment of CD8^+^ T cell immunity. However, VLP-GP33 immunisation failed in old Tx mice, since LCMV titres were between 29- and 1290-fold increased compared to young Tx mice (*p = ns*; Figure 3C, D; open symbols). In addition, old Tx mice with latent MCMV infection showed a trend to even higher LCMV titres in all tested organs (2- to 9-fold, *p = ns*) and thus to reduced protective immunity compared to old Tx mice. Therefore, VLP immunisation was not sufficiently protective against a LCMV-challenge in old Tx mice with and without latent MCMV infection, despite a short interval after VLP priming and challenge with a low dose of a LCMV strain with intermediate virulence.

**Figure 4:**
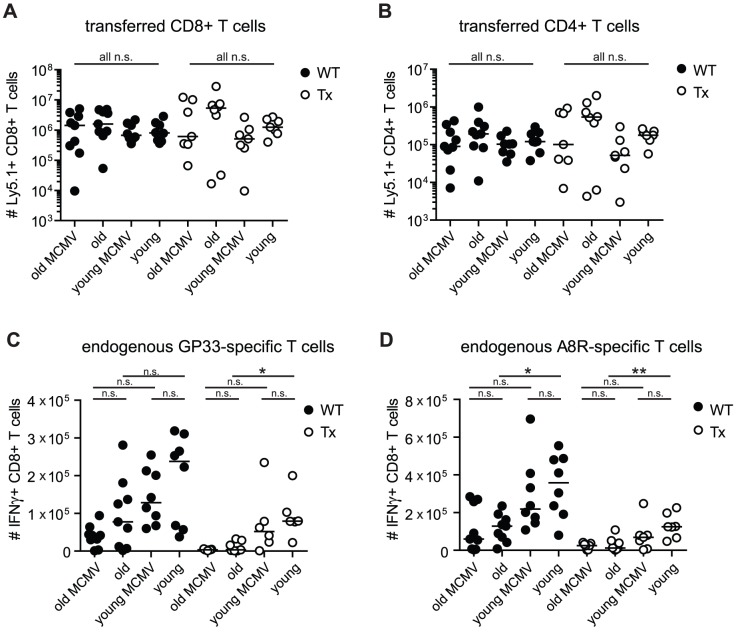
Analysis of virus specific T cell responses after adoptive transfer of TCR transgenic CD8^+^ and CD4^+^ T cells and VACV-GP infection in young and old mice with/without Tx or latent MCMV-infection. Transgenic GP33-specific CD8^+^ and GP61-specific CD4^+^ T cells were isolated from the spleen of young Ly5.1^+^ donor mice and transferred into young (4–6 months old) or old (23–28 months old), MCMV-infected (2–4 or 20–25 months p.i.) or uninfected, Tx (open circles) or non-Tx (wt, closed circles) recipients (Ly5.2). One day later, mice were infected with 5×10^6^ pfu VACV-GP i.p.. Six days later, VACV-GP-specific CD4^+^ and CD8^+^ T cell responses were measured in the lung by tetramer-staining (A) or by ICS after *in vitro* re-stimulation with the respective peptide (B–D) and analysed by flow cytrometry. (A) Total number of GP33-specific transgenic CD8^+^ T cells (Ly5.1) in the lung. (B) Total number of GP61-specific transgenic CD4^+^ T cells (Ly5.1) in the lung. (C) Total number of endogenous GP33-specific CD8^+^ T cells (Ly5.2) in the lung. (D) Total number of endogenous A8R-specific CD8^+^ T cells in the lung. Pooled data from two independent experiments are displayed. Circles indicate values of individual mice, horizontal bars indicate the medians. Significance was assessed by ANOVA followed by Bonferroni post-analysis (* *p*<0.05; ** *p*<0.01; ns = not significant).

To investigate T help dependent antibody responses in these eight groups of mice, the Qβ-specific IgG-response was measured 10 (Figure 4E) and 20 days (Figure 4F) after VLP-GP33 immunisation. In young mice, Qβ-specific IgG-titres were similar at both time points irrespective of Tx and/or latent MCMV-infection. In contrast, Tx and ageing clearly and cumulatively reduced Qβ-specific IgG-responses, particularly on day 10 after immunisation. Qβ-specific IgG-titres were between 5- and 15-fold lower in old/uninfected compared to young/uninfected mice, irrespective of Tx. On day 20, the age-related differences in antibody responses were smaller indicating that ageing alone primarily delayed the response. In contrast, ageing combined with Tx resulted in a delay and an absolute reduction of the Qβ-specific IgG response. Latent MCMV-infection had a small but consistent impact on Qβ-specific IgG-titres in old mice early after immunisation (day 10; Figure 3E, *p = ns*) but not later. Therefore, latent MCMV-infection is rather delaying than suppressing the antibody response after VLP-immunisation. Overall, ageing and Tx clearly reduced the efficacy of VLP-immunisation whereas the influence of MCMV was very limited.

### Expansion of transgenic CD4^+^ and CD8^+^ T cells after VACV-GP infection is not significantly affected by age, thymectomy and latent MCMV-infection

We next investigated whether CD8^+^ T cell intrinsic or extrinsic factors were involved in impaired immune responses of aged, Tx and MCMV-infected mice. To this end, we co-transferred naïve TCR-transgenic (tg) CD8^+^ T cells specific for LCMV-GP33 and TCR-tg CD4^+^ T cells specific for LCMV-GP61 from young Ly5.1 positive mice into the eight different groups of Ly5.2 positive recipient mice mentioned before: young (4–6 months old) or old (23–28 months old); MCMV-infected (for 2–4 months or 21–24 months) or uninfected; Tx (at 4–5 weeks of age) or no Tx. One day later, all recipient mice were infected with 5×10^6^ pfu VACV-GP i.p. Donor and recipient derived GP61-specific CD4^+^ and GP33-specific CD8^+^ T cell responses were quantified in the spleen (data not shown) and in the lung on day 6 after infection. Surprisingly, expansion of TCR-tg CD4^+^ and CD8^+^ T cells was comparable in all groups of recipient mice, although variability within groups was increased in old and in Tx mice (Figure 4A, B). This indicates that MHC class I and class II antigen presentation pathways were sufficiently functional after VACV-GP-infection irrespective of ageing, Tx and latent MCMV-infection to allow comparable expansion of naïve TCR-tg CD4^+^ and CD8^+^ T cell populations.

Interestingly, the endogenous GP33 and A8R-specific CD8^+^ T cell responses showed the familiar pattern: reduced expansion of virus specific CD8^+^ T cells was associated with age, Tx and latent MCMV-infection (Figure 4C, D) but only differences between young and old mice were statistically significant. Despite adoptive transfer of additional and functional LCMV-GP specific TCR-tg CD4^+^ T cells the endogenous GP33-specific CD8^+^ T cell response was not ‘rescued’ in old, old Tx or old MCMV-infected recipients. Since the GP33-specific CD8^+^ T cell response was shown to be T help dependent after VACV-GP-infection [41], this suggests that lack of CD4 T cell help was not a major reason for poor CD8^+^ T cell expansion in these mice. Overall, our findings are best explained by an intrinsic defect of the endogenous CD8^+^ T cell population to expand after cognate antigen encounter due to ageing, Tx and MCMV-infection.

### Impact of age, thymectomy and MCMV-infection on the size and composition of the CD8^+^ T cell compartment

**Figure 5:**
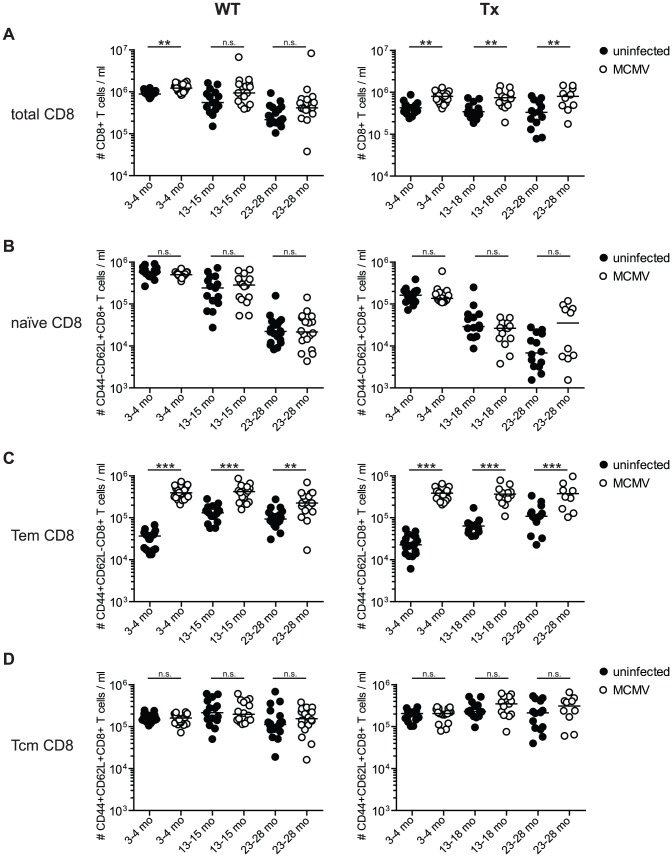
Long-term analysis of the CD8^+^ T cell compartment after MCMV infection and/or thymectomy in young, middle-aged and old mice. C57BL/6 mice were thymectomised at age of 4–5 weeks. Two to 3 weeks later, Tx (right panel) and non-Tx wild type (WT, left panel) mice were infected with 10^7^ pfu MCMV-Δ157 i.v. The absolute number of CD8^+^ T cells was quantified in the blood and phenotypically characterised by polychromatic flow cytometry using CD44 and CD62L. Quantitative analysis of total CD8^+^ T cell numbers (A), naïve CD8^+^ T cell numbers (B; CD8^+^CD44^−^CD62L^+^), effector memory (Tem) CD8^+^ T cell numbers (C; CD8^+^CD44^+^CD62L^−^) and central memory (Tcm) CD8^+^ T cell numbers (D; CD8^+^CD44^+^CD62L^+^). Data were pooled from three independent experiments. Circles indicate values of individual mice, horizontal bars correspond to the median of an experimental group. Significance was assessed by ANOVA followed by Bonferroni post-analysis (* *p*<0.05; ** *p*<0.01; *** *p*<0.001; ns = not significant).

To further investigate the mechanisms behind impaired T cell immunity in the context of ageing, thymic involution (i.e. Tx) and MCMV-infection we performed a detailed quantitative analysis of the impact of these three parameters on the composition of the peripheral T cell pools. There is compelling evidence from human studies that HCMV-infection leaves a unique signature on the CD8^+^ T cell compartment but less so for the CD4^+^ T cell compartment [26]–[28]. We first analysed the CD8^+^ T cell compartment in the blood of young (3–4 months), middle-aged (13–18 months) and old mice (23–28 months) with/without Tx and latent MCMV-infection, respectively (Figure 5).

MCMV-infection of young mice resulted in a substantial expansion of the total CD8^+^ T cell compartment irrespectively of Tx and this difference was maintained during ageing (Figure 5A). Independently of MCMV-infection or Tx, there was an age-associated decline of the total CD8^+^ T cell pool. Finally, Tx initially reduced the size of the total CD8^+^ T cell pool in young and young MCMV-infected mice, but this difference gradually disappeared over time.

**Figure 6:**
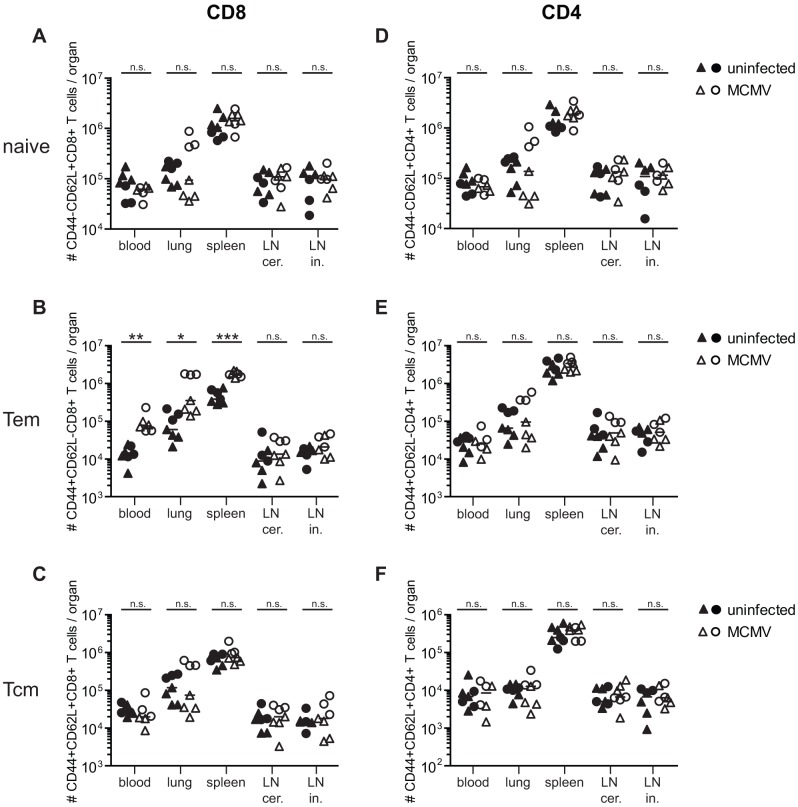
Alterations of CD4^+^ and CD8^+^ T cell populations in secondary lymphoid and non-lymphoid organs of middle-aged mice with (open symbols) and without (closed symbols) latent MCMV-infection. Half of the mice were infected with 10^7^ pfu MCMV-Δ157 i.v. at the age of 8 weeks. At the age of 8 (triangles) or 13 (circles) months, blood was taken, mice were perfused and sacrificed and total numbers of naïve (CD44^−^CD62L^+^) CD8^+^ (A) and CD4^+^ (D) T cells, effector memory (Tem; CD44^+^CD62L^−^) CD8^+^ (B) and CD4^+^ (E) T cells and central memory (Tcm; CD44^+^CD62L^+^) CD8^+^ (C) and CD4^+^ (F) T cells were determined in different organs by polychromatic flow cytometry using bead calibration. Absolute numbers of T cells are given for blood, lung, spleen, cervical (cer) and inguinal (in) lymph nodes (LN). Circles indicate values of individual mice, horizontal bars correspond to the median of an experimental group. Significance was assessed by t-test (* *p*<0.05; ** *p*<0.01; *** *p*<0.001; ns = not significant).

Several human studies have demonstrated that HCMV infection is associated with a sizeable reduction of the naïve CD8^+^ T cell pool, although most of these studies recorded T cell frequencies and not total numbers [26], [28]. Indeed, our results confirm that MCMV-infection is associated with an immediate and pronounced reduction of naïve CD8^+^ T cell frequencies comparable to human studies (Figure S3A). To our surprise, absolute numbers of naïve CD8^+^ T cells were similar in the blood of uninfected and MCMV-infected mice (Figure 5B). These data were confirmed in another cohort of 8 and 13 months old mice, where absolute naïve CD8^+^ T cell numbers were not reduced by latent MCMV-infection in non-lymphoid (lung, blood) and in secondary lymphoid organs (spleen, cervical and inguinal lymph nodes, Figure 6A). In contrast, ageing and Tx clearly and cumulatively reduced the total naïve CD8^+^ T cell pool leaving very few naïve T cells in old Tx mice. We suspect that the apparent increase of naïve T cells in old/Tx/MCMV-infected mice (Figure 5B, right panel, right column) might be caused by a poor discrimination of CD44-high and CD44-low T cell populations in this group of mice.

In contrast to the naïve T cell pool, MCMV-infection induced a massive (>10-fold) expansion of blood CD44^+^ CD62L^−^ effector memory (Tem) CD8^+^ T cells in young mice independent of Tx (Figure 5C). During ageing, Tem-numbers slowly increased in uninfected and uninfected Tx mice and remained stable in MCMV-infected mice. Although the impact of MCMV-infection on Tem differences gradually dwindled with increasing age, Tem-numbers were consistently and significantly increased by MCMV-infection across all age groups, whereas Tx had no significant influence. Tem-numbers were also significantly increased in the lung and spleen of MCMV-infected mice whereas numbers were comparable to MCMV-naïve mice in both sets of lymph nodes (Figure 6B).

Contrary to Tem, the CD44^+^ CD62L^+^ central memory (Tcm) CD8^+^ T cell pool was not measurably altered by MCMV-infection in blood, lung, spleen or lymph nodes (Figure 5D and 6C). In addition, neither ageing nor Tx had a measurable influence on absolute numbers of blood Tcm (Figure 5D). In summary, ageing and Tx mainly reduced the naïve CD8^+^ T cell pool whereas MCMV-infection had surprisingly little influence on naïve T cell numbers but massively boosted the Tem pool. These additional T cells were accommodated in the CD8-compartment by a long lasting expansion of the total CD8^+^ T cell pool in MCMV-infected mice and not by a reduction of the naïve T cell pool.

### Impact of age, thymectomy and MCMV-infection on the size and composition of the CD4^+^ T cell compartment

**Figure 7:**
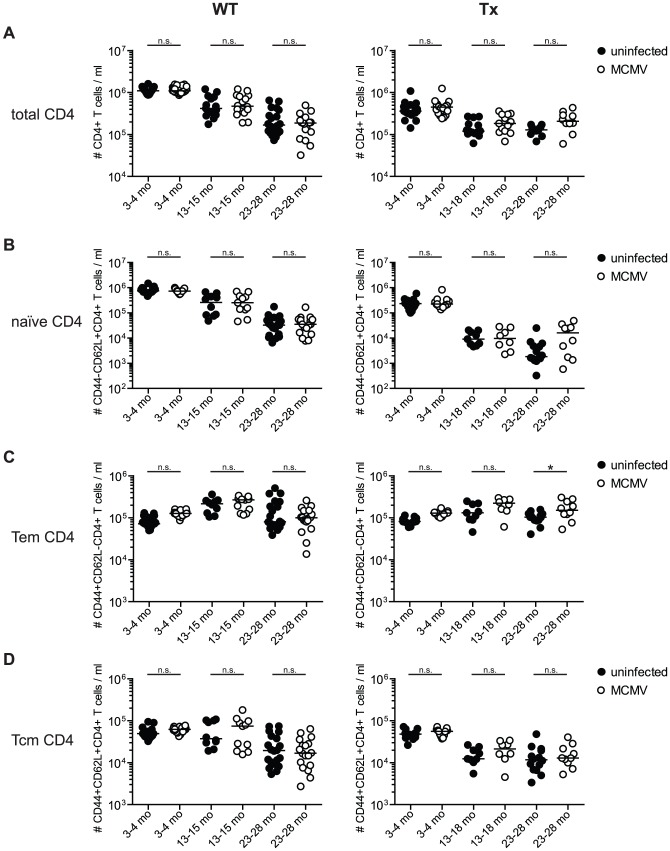
Long-term analysis of the CD4^+^ T cell compartment after MCMV infection and/or thymectomy in young, middle-aged and old mice. C57BL/6 mice were thymectomised at age of 4–5 weeks. Two to 3 weeks later, Tx (right panel) and non-Tx wild type (WT, left panel) mice were infected with 10^7^ pfu MCMV-Δ157 i.v. The absolute number of CD4^+^ T cells was quantified in the blood and phenotypically characterised by polychromatic flow cytometry using CD44 and CD62L. Quantitative analysis of total CD4^+^ T cell numbers (A), naïve CD8^+^ T cell numbers (B; CD4^+^CD44^−^CD62L^+^), effector memory (Tem) CD4^+^ T cell numbers (C; CD4^+^CD44^+^CD62L^−^) and central memory (Tcm) CD4^+^ T cell numbers (D; CD4^+^CD44^+^CD62L^+^). Data were pooled from three independent experiments. Circles indicate values of individual mice, horizontal bars correspond to the median of an experimental group. Significance was assessed by ANOVA followed by Bonferroni post-analysis (* *p*<0.05; ns = not significant).

The total CD4^+^ T cell compartment was not significantly altered by MCMV-infection (Figure 7A) although, there was a trend to increased CD4^+^ T cell numbers in Tx mice with latent MCMV-infection. In contrast, both age and Tx significantly and cumulatively reduced the total CD4^+^ T cell pool. Similarly, latent MCMV-infection did not measurably influence the total number of naïve CD4^+^ T cells in the blood (Figure 7B) or in different lymphoid and non-lymphoid organs (Figure 6D). Ageing was associated with a progressive but gradual loss of naïve T cell numbers whereas the very prominent Tx-associated reduction appeared within 6 months after Tx, leading to a >30-fold drop of naïve CD4^+^ T cell numbers from young to middle-aged mice (Figure 7B).

In the memory compartment, latent MCMV-infection also led to a slight expansion of blood CD4^+^ Tem but not of Tcm in young mice and this difference was partially maintained for Tem during ageing (Figure 7C, D). However, in lung, spleen and lymph nodes CD4^+^ Tem and Tcm numbers were similar in MCMV-infected and MCMV-naïve mice (Figure 6E, F). Ageing and Tx only had a minimal influence on the total number of CD4^+^ Tem (Figure 7C). Ageing led to a gradual and progressive reduction of CD4^+^ Tcm in normal mice whereas this process was accelerated in Tx-mice leading to low Tcm numbers already in middle-aged mice (Figure 7D). Overall, the effects of MCMV-infection on the CD4-compartment were much more subtle but similar to CD8 as an overall trend, whereas Tx had a more profound and immediate impact on the total and the naïve CD4 compartment.

## Discussion

Our data demonstrate for the first time in a mouse model that long-term latent MCMV-infection interferes with the induction of protective immunity in aged mice. Latent MCMV-infection additively impaired the poor control of LCMV-infection in old mice and this loss of antiviral protection was linked to reduced expansion of LCMV-specific CD8^+^ T cells. Moreover, latent MCMV-infection was associated with decreased CD8^+^ T cell expansions in old mice after infection with VACV but not after VLP-immunisation. Nevertheless, protective efficacy of VLP-immunisation against LCMV-challenge was impaired in old Tx mice with latent MCMV-infection. These data from a variety of infectious and non-infectious experimental models establish a causal role of latent MCMV-infection as a propagating factor for poor immunity in old age contributing to T cell based immune senescence. Our data are strongly corroborated by similar results from Cicin-Sain et al., who found reduced CD8^+^ T cell responses after superinfection with Influenza virus, West Nile virus (WNV) and Herpes simplex virus Type 1 in old mice with latent MCMV-infection [42].

At first glance our findings seem to contradict an earlier study demonstrating partial protection against bacterial challenge in mice recently infected with MCMV or murine herpesvirus-68 by activated innate immune responses [43]. Although our study primarily focused on the impact of long-lasting latent MCMV-infection on T cell immunity in old mice, where we demonstrate impaired T cell mediated antiviral protection in latently infected mice, we have neither seen any protective effect of MCMV-infection against LCMV or VACV in young mice (Figure 1A, B; VACV not shown). These discrepancies are most likely explained by differences in challenge systems: while Barton et al. used bacterial pathogens (i.e. Listeria and Yersinia) [43] we and Cicin-Sain have applied viral challenges [42]. Alternatively, partial protection by activated innate immune mechanisms may have been counteracted by impaired adaptive immunity in young mice with latent MCMV-infection.

Our findings in this experimental mouse model strongly support previous observational human studies suggesting that HCMV-infection may be associated with accelerated immune senescence [12]. This notion was largely based on longitudinal cohort studies and cross-sectional epidemiological studies demonstrating a significant association of HCMV-seropositivity with decreased survival of very elderly [13]–[15]. Moreover, HCMV-infection was associated with reduced immunogenicity of influenza vaccination in elderly [17] although this was not confirmed more recently [16]. Interestingly, immunogenicity of CpG-adjuvanted VLPs was sufficient to largely overcome the age- and MCMV-associated immune attenuation in our experimental model (Figure 3). Only very old Tx mice with particularly low naïve T cell counts before VLP-immunisation were not protected against a LCMV-challenge (Figure 3C, D) This may indicate that more immunogenic vaccines than those currently available for influenza could help to improve protective immunity in the subset of elderly with a minimally maintained naïve T cell repertoire.

All of the above mentioned human studies about the impact of naturally acquired HCMV-infection on waning immunity in the elderly are limited by the fact that a variety of factors may strongly influence any significant association. Potential confounders include 1) individual characteristics of HCMV-infection like virus isolate, dose, route, time point and severity of primary infection and the frequency of re-infection or re-activation, 2) genetic variability of the host directing the antiviral immune response, 3) general health and socio-economic situation of the host both presently and in the past (co-morbidities, medication, nutrition, living conditions, physical activity, health seeking behaviour a.s.o.), and 4) the host's past infectious history apart from HCMV. For obvious reasons, it is impossible to control for most of these parameters in human studies. In contrast, we were able to exclude or balance these confounders in our mouse model. Therefore, our data provide the most direct evidence that CMV-infection itself is the driving force for the association between CMV-infection and impaired immunity in aged hosts.

Concerning the major mechanism of CMV-enhanced immune senescence, most experts in the field favour the concept that CMV-driven memory T cell accumulations progressively restrict the size and the diversity of the naïve T cell pool, thus exacerbating the age associated alterations of the T cell compartment [44]. Studies in old mice and macaques have clearly established that both size and diversity of the naïve T cell pool are crucial for maintenance of immune protection [11], [45]. Indeed, several human studies have shown that naïve CD8^+^ T cell frequencies in the blood were significantly reduced in HCMV-infected elderly or thymectomised young adults [26], [28], [46] but only few studies have reported significant reductions of absolute naïve CD8^+^ T cell numbers [27]. Our data demonstrate that total numbers of naïve CD4^+^ and CD8^+^ T cells were not significantly reduced by MCMV-infection in blood, spleen, lymph nodes and lung. This argues against the currently favoured hypothesis that CMV-infection reduces the number of available naïve T cells. At present, we cannot formally exclude the possibility of MCMV-dependent qualitative alterations of the naïve T cell pool, especially concerning repertoire diversity.

Since adoptively transferred CD4^+^ and CD8^+^ T cells displayed comparable immune responses in all recipients irrespective of ageing, thymectomy and MCMV-infection, our results are best explained by a deficient activation and/or expansion of the endogenous T cell population (Figure 4). Indeed, Cicin-Sain et al were able to demonstrate an inhibitory effect of latent MCMV-infection on T cell recruitment and/or activation exclusively in draining lymph nodes after intranasal Influenza infection [42]. Our data further suggest that MHC class I and class II dependent antigen presentation was not substantially altered by ageing, thymectomy or latent MCMV-infection to interfere with activation and expansion of tg CD4^+^ and CD8^+^ T cells after VACV-infection. This is in line with similar antigen presentation in young and old monkeys after Vaccinia immunisation [45]. Although productive MCMV-infection has been shown to severely impair DC function [47] our results argue against a major interference of latent MCMV-infection with overall functionality of DCs as an explanation for reduced heterologous antiviral immunity: 1) Latent MCMV-infection of young mice was not associated with any measurable reduction of T cell immunity. 2) Activation, expansion and functionality of adoptively transferred naïve transgenic CD4^+^ and CD8^+^ T cells were comparable in all hosts tested, irrespective of MCMV-infection, age and Tx (Figure 4A, B). 3) Latent MCMV-infection did not significantly impair the primary CD8^+^ T cell response after VLP-GP33 immunisation in old mice (Figure 3A) although protective efficacy was eventially reduced in MCMV-infected old Tx mice (Figure 4C, D). Moreover, Cicin-Sain et al show that latent MCMV-infection with a mutant virus deficient for critical immune evasive genes was still associated with poor WNV-specific T cell immunity [42]. Lastly, it is unlikely that limiting T cell help was a major factor for MCMV-infection associated immune failure in our experimental setup. Provision of additional T cell help by adoptive transfer of tg CD4^+^ T cells was not sufficient to rescue the poor endogenous CD8^+^ T cell response in old, Tx and MCMV-infected mice although CD8^+^ T cell expansion after VACV-GP infection is known to be help dependent [41].

As opposed to the naïve T cell compartment, there is compelling evidence in mice, macaques and humans that CMV-infection drastically increases the memory T cell pool, particularly regarding CD8^+^ Tem [19], [25]–[28], [48]–[53]. Interestingly, those human studies indicating an association of HCMV-infection with poor vaccine immunogenicity or with reduced survival of very elderly, have also reported an association with CD8^+^ Tem expansions and not with a reduction of naïve T cells [15], [17]. Similarly, the correlation of poor T cell expansion after heterologous WNV infection was stronger for increased Tem than for reduced naïve T cells in the MCMV-model [42]. Therefore, data from the MCMV-model support the human findings and demonstrate a vigorous expansion of Tem after MCMV-infection which was maintained into old age by memory inflation. MCMV-dependent Tem accumulation caused a significant and long lasting expansion of the total CD8^+^ T cell pool without restricting the available immunologic ‘space’ for naïve T cells explaining the discrepancy between reduced frequencies but maintained total numbers of naïve T cells after MCMV-infection (Figure 5B, S3A). This highlights the fact that the CD8^+^ T cell compartment has considerable plasticity and can grow in size according to the cumulative antigen experience of the host [54].

**Figure 8:**
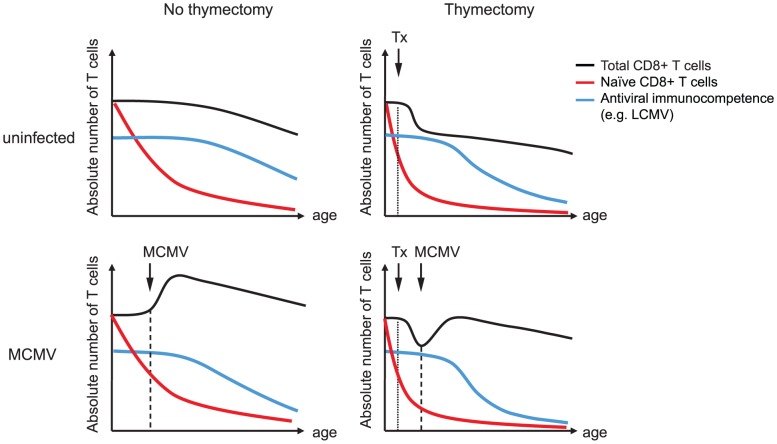
Integrative model of the influence of MCMV-infection, ageing and thymectomy on CD8^+^ T cell populations and protective immunity. Ageing is accompanied by a slow but progressive decline of the total (black line) and the naïve (red line) CD8^+^ T cell pool. Immune senescence is characterised by an age-associated reduction of immunocompetence (blue line), particularly against newly encountered antigens (e.g. LCMV). Tx of young mice leads to a substantial and rapid shrinking of the naïve T cell compartment reducing the number and the diversity of naïve T cell precursors on top of ageing. Tx also reduces the total CD8^+^ T compartment, which is later affected by an age-associated decline. MCMV-infection results in an immediate and persistent expansion of the total CD8^+^ T cell compartment whereas the naïve T cell pool remains intact. Nevertheless, MCMV-infection contributes to reduced immunocompetence over time, possibly by an enhanced competition of massively expanded CMV-specific Tem with newly generated effector cells. In old Tx mice with latent MCMV-infection, immuocompetence is cumulatively affected by the restricted naïve T cell pool and by the increased competition of CMV-specific Tem.

Based on these findings, we postulate that CMV-dependent CD8^+^ Tem expansion is more important for CMV-enhanced immune senescence than naïve T cell suppression. Moreover, we would like to propose a model of enhanced competition for immunologic niches and survival factors between pre-existing CMV-specific memory T cells and newly generated effector T cells after heterologous infection or immunisation (Figure 8). CMV maintains memory T cells at very high frequencies well into old age, preferentially in the CD8^+^ T cell compartment as Tem, which persistently occupy critical niches both within secondary lymphoid (i.e. spleen) and peripheral organs. At first glance, the absence of Tem accumulations in lymph nodes of MCMV-infected mice (Figure 6C, D) may contradict a competitive model. However, Torti et al have recently shown that MCMV-specific inflationary T cells maintain a Tcm phenotype in lymph nodes where they interact with antigen presenting cells of non-hematopoietic origin and then egress the lymph nodes probably as Tem [55]. Therefore, competition of expanded MCMV-specific memory T cell populations with newly generated effector cells after heterologous infection or immunisation may still take place within, on the way out or even outside lymph nodes. Of note, we find the most robust differences in T cell immunity after systemic LCMV-infection (Figure 1A–D), where T cell competition may preferentially occur in the spleen and not in lymph nodes. Moreover, LCMV-infection induces a massive inflammatory response which has the potential to trigger MCMV-reactivation. If this occurs expanded CMV-specific memory T cell populations are not only induced by bystander activation but also directly by TCR-mediated signals leading to further memory inflation and potentially to enhanced competition with heterologous T cell responses. However, our hypothesis of enhanced T cell competition needs to be tested in future studies together with a detailed analysis of the naïve T cell repertoire in old mice with and without latent MCMV-infection, since the absolute number of naïve precursor T cells is most likely to be CMV-independent but nevertheless crucial for maintained immunity in aged hosts. This is suggested by our findings of comparable T cell immunity in latently infected young mice, where the T cell repertoire was not yet affected by ageing or Tx, and supported by the comparable expansion of tg T cells irrespective of age, MCMV-infection or Tx, where we artificially increased and normalized the naïve precursor frequency.

In summary, our results demonstrate that MCMV-infection has a moderate but significant negative influence on antiviral immune responses and protective immunity in old mice. However, Tx at young age and ageing itself seem to restrict immunity more profoundly than MCMV-infection. The involved mechanisms are likely to be different since ageing and Tx operate via the restriction of the naïve T cell pool while latent MCMV infection leaves the naïve T cell pool untouched but mainly propagates memory and total T cell expansions (Figure 8). Our model of latent MCMV-infection with or without Tx seems to be well suited to further investigate the underlying mechanisms and to develop and test preventive strategies. Finally, our results strongly support previous human studies that have found an association of CMV-infection with declining immunity in old age [12]. Since we and others were able to demonstrate a similar association in a very well controlled mouse model [42] there is now solid evidence that latent CMV-infection is a propagating factor for immune senescence in mice and in humans.

## Materials and Methods

### Ethics statement

This study was carried out in strict accordance to the guidelines of the animal experimentation law (SR 455.163; TVV) of the Swiss Federal Government. The protocol was approved by Cantonal Veterinary Office of the canton of Zurich, Switzerland (Permit number 174/2006 and 158/2010). Surgery was performed under isoflurane anesthesia and all efforts were made to minimize suffering.

### Mice

C57BL/6 mice were purchased from Jackson Laboratory (Germany) and Charles River (Germany) and were kept under specific pathogen free (SPF) conditions throughout the study. Transgenic mice expressing the TCR specific for the LCMV-GP derived CD8^+^ T cell epitope GP33 (P14 mice) and mice expressing the TCR specific for the LCMV-GP derived CD4^+^ T cell epitope GP61 (Smarta mice) were provided by Prof. A. Oxenius (ETH Zurich, Switzerland) [56], [57]. C57BL/6 mice were thymectomised at 4–5 weeks of age by vacuum extraction of the thymus along the trachea. Mice were anesthetized with isoflurane during the procedure and received analgesia with Buprenorphin i.p. from day -1 until day 6 after Tx. They entered the experiment after a recovery period of 2–3 weeks.

### Viruses and virus like particles

All viruses were provided by Prof. A. Oxenius (Zurich, Switzerland). The recombinant MCMV-Δ157 is a deletion mutant of MCMV lacking the m157 ORF leading to decreased NK-cell control in C57BL/6 mice [58]. The mutant virus was originally generated according to the published method using the bacterial artificial chromosome BAC pSM3fr [59]. MCMV was propagated on mouse embryonic fibroblasts (MEFs) and virus titres of virus stocks and organ homogenates were determined by plaque assays on MEFs as previously described, using centrifugal enhancement of infectivity and an adapted Avicel overlay (3% Avicel, 10% MEM, 10% FCS, 200 mM Glutamine, penicillin, streptomycin, 10 mM HEPES/NaHCO_3_, H_2_O) [60]. Mice were infected with 10^7^ pfu of MCMV-Δ157 at the age of 6–10 weeks. LCMV-WE was propagated on L929 fibroblast cells. LCMV titres were determined by a virus focus forming assay on MC57G fibroblasts as previously described [61]. Mice were infected with 2×10^3^ pfu LCMV-WE i.v. at the indicated time points. VACV-GP was propagated and plaqued on BSC40 cells. Mice were infected with 5×10^6^ pfu VACV-GP i.p. at the indicated time points. The virus like particles VLP-GP33 were composed of the core protein of the Qβ-bacteriophage, coupled with the LCMV derived GP33-peptide, packaged with CpG oligonucleotides (5′-GGGGTCAACGTTGAGGGGGG-3′, thioester stabilized) and produced as previously described [40]. Mice were immunized with 200 µg VLP-GP33 s.c. into the flank at the indicated time points. The VLPs were provided by Dr. M. Bachmann (Cytos Biotechnology, Schlieren, Switzerland).

### Adoptive transfer

Adoptive transfer experiments were performed with cells isolated from spleens of tg Ly5.1-positive P14 or Smarta mice into Ly5.2 positive recipients to allow for simple discrimination of their origin by FACS. Using CD8 or CD4-beads, TCR-tg CD8^+^ or CD4^+^ T cells were purified by MACS from donor P14 mice or Smarta mice, respectively. 10^4^ naïve tg CD8^+^ and CD4^+^ T cells were mixed and co-transferred i.v. into naïve or MCMV-infected recipient mice. One day later, mice were infected with 5×10^6^ pfu VACV-GP i.p. and responses were analysed after six days.

### Isolation of murine lymphocytes from solid organs

Infected or uninfected mice were anesthetized i.p. with an anaesthetic cocktail containing Xylazin (Rompun), Ketamin (Ketasol) and Acepromazin (Prequillan). After the surgical level of anaesthesia was reached mice were perfused with 5–10 ml of cold PBS to remove all contaminating blood from the organs. Organs were collected, cut into small pieces and digested by incubating for 20 min at 37°C with 3 ml of an enzyme cocktail (x100 stock: 2.4 mg/ml collagenase and 0.2 mg/ml DNAse dissolved in RPMI medium with 10% FCS). After gentle mechanical tissue disruption by pulling the sample through an 18 G needle and additional 20 min incubation with 2 ml of fresh enzyme cocktail the sample was pushed through a cell strainer and centrifuged. After a Percoll gradient centrifugation to isolate viable lymphocytes the cells were washed twice, counted and resuspended in RPMI 10% FCS for further analysis. Single cell suspensions of spleen cells were produced by gently pressing the organ trough a grid of stainless steel with a plug. Viable cells were counted by trypan blue exclusion using Neubauer counting chambers.

### Antibodies and tetramers

Monoclonal antibodies for flow cytometry assays were purchased from Becton Dickinson (Switzerland) or BioLegend (Switzerland). The following antibodies were used: anti-CD8-Pacific Blue/PerCp (clone 53-6.7), anti-CD4-PE (clone RM4–5), anti-CD44-PE-Cy7 (clone IM7), anti-CD62L-FITC (clone MEL-14), anti-IFNγ-APC/PE-Cy7 (clone XMG1.2), and anti-CD45.1-PE (Ly5.1) (clone A20).

M38-/M45-specific (MCMV) and GP33-specific (LCMV) CD8^+^ T cells were detected by MHC class I tetramer staining using APC-conjugated tetramers. Tetramers were produced as previously published [62].

### Immunofluorescence staining and analysis

50–100 µl of blood or 10^6^ cells isolated from spleen or lung were stained with fluorochrome conjugated monoclonal antibodies for 20 min at 4°C. Tetramer staining was performed for 20 min at 4°C or 37°C. Using BD FACS Lysing Solution cells were fixed for 10 min at RT. After a washing step, conjugated antibodies were added to the cells for 20 min at 4°C and cells were washed and resuspended in 200 µl FACS buffer for analysis (PBS, 5 mM EDTA, 2% FCS, 0.05% NaN_3_). Samples were measured with a 6 or 8 colour BD FACS Canto II Flow Cytometer using FACS Diva software. The data files were analysed with Flowjo version 7.5.2. Calibration with beads was used to measure and calculate total cell numbers.

### Peptides and in vitro stimulation of CD8^+^ T cells

MCMV derived peptides M45_985–993_ (HGIRNASFI, H2-D^b^) and M38_316–323_ (SSPPMFRV, H2-K^b^), LCMV-derived peptide GP33_33–41_ (KAVYNFATC, H2-D^b^), NP396_396–404_ (FQPQNGQFI, H2-D^b^) and Vaccinia derived peptides B8R_20–27_ (TSYKFESV, H2-K^b^), A3L_270–277_ (KSYNYMLL, H2-K^b^) and A8R_189–196_ (ITYRFYLI, H2-K^b^) were purchased from EMC Microcollections (Tübingen, Germany). Cytokine production of activated cells was detected by intracellular cytokine staining. 2×10^6^ tissue lymphocytes or splenocytes were stimulated with 10^−6^ M specific peptide for 5–6 h at 37°C in the presence of Monensin (2 µM) or/and Brefeldin A (10 µg/ml). Cells were then washed with FACS buffer and stained for cell surface markers for 20 min at 4°C. Cells were fixed and permeabilized with BD FACS Lysing Solution (1∶5 dilution, 0.05% Tween20) for 10 min at RT and washed with FACS buffer. Cytokine specific antibodies were added to the cells for 20 min at 4°C. After washing cells were resuspended into 200 µl PBS with 1% paraformaldehyde and acquired with a FACS Canto II cytometer.

### ELISA

Qβ-specific IgG antibodies were quantified by ELISA as previously described [63].

### Statistical analysis

One-way ANOVA followed by Bonferroni post-analysis was used for group comparisons using Graph Pad Prism (GraphPad Software, La Jolla, CA). The p-values are indicated in the graphs (*<0.05, ** p<0.01, *** p<0.001).

## Supporting Information

Figure S1 A3L and A8R-specific T cell responses after VACV-GP33-infection of young and old mice with and without latent MCMV-infection. Young (4 months old, 2 months p.i.) and old C57BL/6 mice (25 months old, 23 months p.i.) with and without latent MCMV-infection were infected with 5×10^6^ pfu VACV-GP i.p. At the peak of the response on day 6, A3L-specific (A) and A8R-specific (B) CD8^+^ T cells from the lung were quantified by ICS after *in vitro* re-stimulation with the respective peptide. Circles indicate total numbers of epitope specific CD8^+^ T cells of individual mice, horizontal lines show the medians of an individual group. ANOVA followed by Bonferroni post-analysis was used to determine significant differences between the displayed medians of each group (* *p*<0.05; ** *p*<0.01; *** *p*<0.001; ns = not significant).
(EPS)

Figure S2 MCMV control in thymectomised mice. C57BL/6 mice were thymectomised at age of 4–5 weeks. Two to 3 weeks later, Tx and non-Tx wild type (WT) mice were infected with 10^7^ pfu MCMV-Δ157 i.v. Eight days after MCMV infection viral titres were determined by plaque assay in salivary glands (SG), spleen, lung and liver. One of two similar experiments is shown. Circles indicate values of individual mice, horizontal bars correspond to the median of an experimental group. T-test was used to determine significant differences between the displayed medians of respective groups (ns = not significant).
(EPS)

Figure S3 Long-term analysis of naïve CD4^+^ and CD8^+^ T cell frequencies after MCMV infection and/or thymectomy in young, middle-aged and old mice. C57BL/6 mice were thymectomised at age of 4–5 weeks. Two to 3 weeks later, Tx (right panel) and non-Tx wild type (WT, left panel) mice were infected with 10^7^ pfu MCMV-Δ157 i.v. The frequencies of naïve CD8^+^ and CD4^+^ T cells were quantified in the blood by polychromatic flow cytometry using CD44 and CD62L. Frequencies of naïve CD44−, CD62L^+^ CD8^+^ (A) and CD4^+^ (B) T cells are shown. Data from three independent experiments also shown in Figure 5 and 7 were pooled. Circles indicate values of individual mice, horizontal bars correspond to the median of an experimental group. Significance was assessed by ANOVA followed by Bonferroni post-analysis (* *p*<0.05; *** *p*<0.001; ns = not significant).
(EPS)
